# ﻿A taxonomic review of the genus *Fignya* Solovyev & Witt, 2009 (Lepidoptera, Limacodidae) with descriptions of three new species and one new combination

**DOI:** 10.3897/zookeys.1213.132318

**Published:** 2024-09-26

**Authors:** Jun Wu, Huilin Han

**Affiliations:** 1 School of Forestry, Northeast Forestry University, Harbin 150040, China Northeast Forestry University Harbin China; 2 Northeast Asia Biodiversity Research Center, Northeast Forestry University, Harbin 150040, China Northeast Forestry University Harbin China; 3 Ministry of Education, Key Laboratory of Sustainable Forest Ecosystem Management, Northeast Forestry University, Harbin 150040, China Northeast Forestry University Harbin China

**Keywords:** China, identification key, morphology, new combination, slug caterpillar moths, taxonomy, Zygaenoidea

## Abstract

Three new species of the genus *Fignya* Solovyev & Witt, 2009, *F.qiana***sp. nov.**, *F.trigonum***sp. nov.**, and *F.samkosa***sp. nov.**, are described from China and Cambodia. Additionally, a new combination, *F.brachygnatha* (Wu & Fang, 2008), **comb. nov.**, is proposed. The new species are illustrated with images of the adults and male genitalia, and compared with similar species. A key to all known species in the genus, along with their geographical distributions, is provided.

## ﻿Introduction

The genus *Fignya* is a recently established small genus within the family Limacodidae. Currently, only two species are included in this genus: *F.melkaya* Solovyev & Witt, 2009 and *F.ravalba* Wu, Solovyev & Han, 2022. The type species, *F.melkaya*, was first discovered in northern Vietnam on Mt. Fan-si-pan (West) and later also found in Sichuan, China; *F.ravalba* was discovered in Medog County, Xizang Autonomous Region, China, and is so far only known from its type locality (Ji 2018; Wu et al. 2022).

The diagnostic characteristics of this genus have been thoroughly described in previous studies: small in size, filiform antennae in both sexes; slightly up-curved labial palpi; and a large white spot on the forewing in the Cu area with a brown border. The forewing also features a sinusoidal vein R_1_, with veins R_3_+R_4_ branched from R_5_. The tibial spurs are in a 0-2-4 formula. In the male genitalia, the gnathos is fishtail-shaped with a comb-like apex; and the vesica bears large, strongly sclerotized cornuti (Solovyev and Witt 2009; Wu et al. 2022).

In this study, we propose a minor expansion of the definition of this genus, focusing mainly on the male genitalia: (1) the gnathos is fishtail-shaped with a comb-like apex, or the gnathos is spoon-shaped, with a weakly sclerotized, nearly membranous apex densely covered with small scobinations; (2) the base of the valva usually has an obvious, large, hairy process which bears large lateral bristles, though the process may be reduced or absent in some taxa; and (3) the vesica bears large, strongly sclerotized cornuti, or the phallus is bifurcated with a pointed spine terminally.

During the examination of moths from southwestern China and Cambodia, we identified several specimens of *Fignya*. While these specimens morphologically resemble the two known congeners, dissection revealed significant differences in the structure of the male genitalia. Consequently, we formally describe them as new species, increasing the number of species within *Fignya* to six. A diagnosis, illustrations, and a key to species with distributions are also provided.

## ﻿Materials and methods

The type series was collected with a 220V/450W mercury vapor light and a DC black light in China and Cambodia. Standard methods for dissection and preparation of the genitalia slides were used (Kononenko and Han 2007). The specimens were photographed using a Canon M6 II camera, whereas the genitalia slides were photographed with an Olympus photo microscope aided by Helicon Focus 7 software and further processed in Adobe Photoshop CS6. Terminology of genitalia follows Epstein (1996) and Kristensen (2003).

All type materials of the new species are deposited in the collection of the Northeast Forestry University (NEFU), Harbin, China.

Abbreviations used:

**IZCAS**Institute of Zoology, Chinese Academy of Sciences, Beijing, China

**MWM/ZSM** Museum Witt München/Zoologische Staatssammlung München, Munich, Germany

**NEFU** Northeast Forestry University, Harbin, China

**HT** Holotype

**PT** Paratype

## ﻿Taxonomic account

### ﻿Genus Fignya

Taxon classificationAnimaliaLepidopteraLimacodidae

Solovyev & Witt, 2009

7AB6ACDD-B649-5D27-9BBB-6B6D3AA6B53E

Fignya Solovyev & Witt, 2009: 197. Type species (by original designation): Fignyamelkaya Solovyev & Witt, 2009. Type locality: Vietnam, Mt. Fan-si-pan (West).

### ﻿ Fignya qiana sp. nov.

Taxon classificationAnimaliaLepidopteraLimacodidae

56434687-F9FF-5F0B-94C5-5BF55C0EA8F6

https://zoobank.org/42F53B62-6D7A-4E42-B089-95704BC8A17D

**Figures 1–6.**
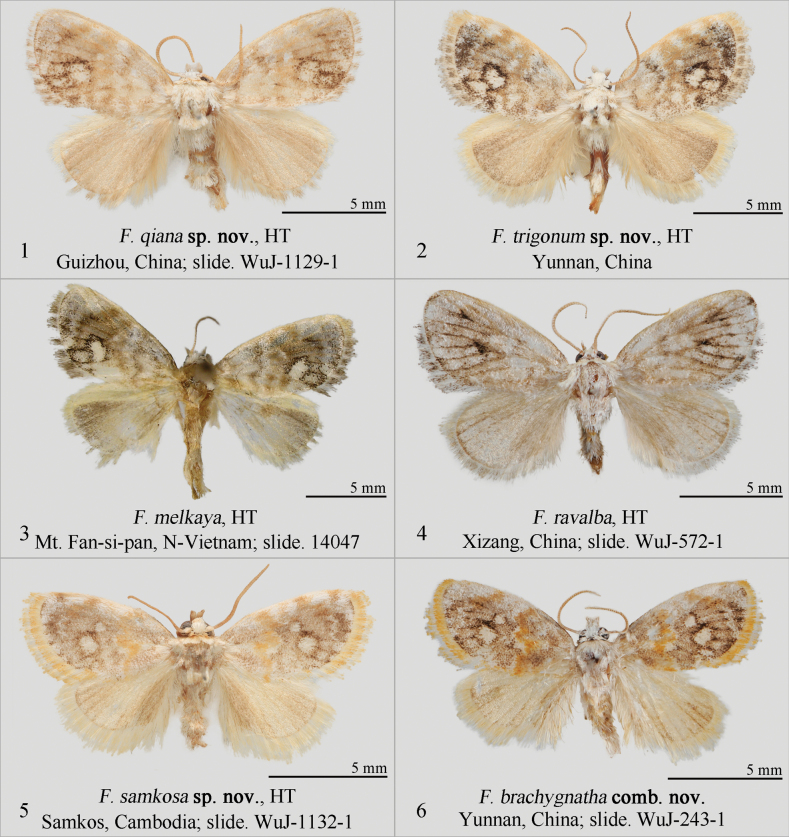
Adults of *Fignya* spp. Depositories of the specimens **1–2, 4–6** in NEFU**3** in MWM/ZSM. Scale bars: 5 mm.

**Figures 7–15.**
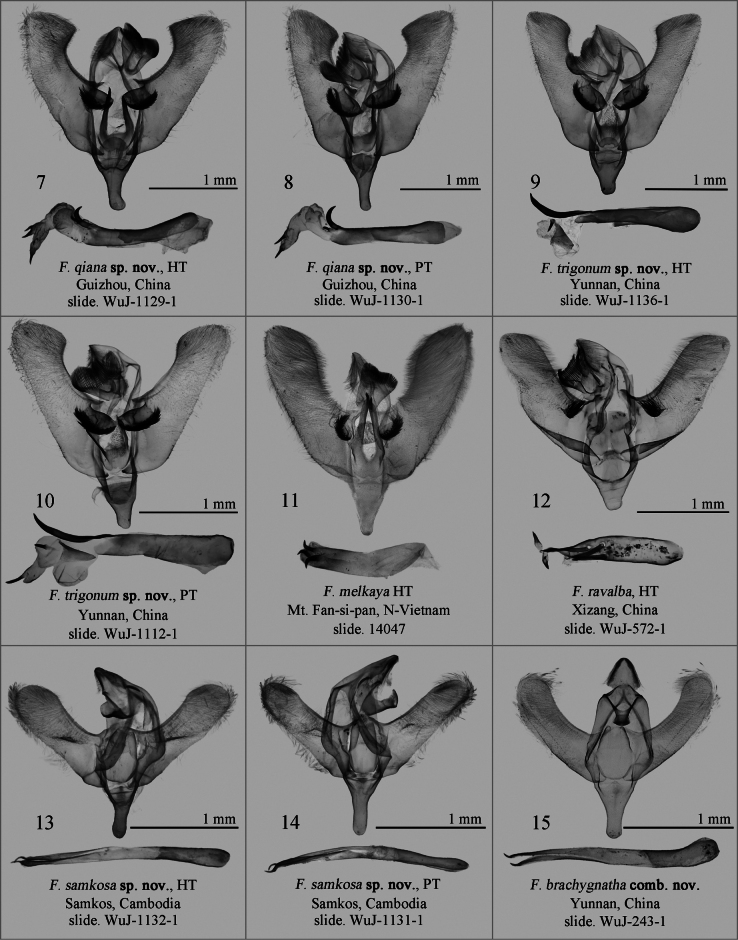
Male genitalia of *Fignya* spp. Depositories of the slides **7–10, 12–15** in NEFU**11** in MWM/ZSM. Scale bars: 1 mm, except unscaled Fig. 11.

Figs 1 , 7 , 8 , 18 , 19

#### Type material.

***Holotype***: China • ♂, Guizhou Province, Xingren City, Linggangshang, 1470 m, 29.vi.2022, J Wu, B Gao & RT Xu leg., genit. prep. WuJ-1129-1, in NEFU.

***Paratypes*** • 3 ♂, same data as for holotype, genit. prep. WuJ-806-1, 1130-1, all in NEFU.

#### Diagnosis.

The new species is not noticeably different in appearance from *F.melkaya* (Fig. 3), *F.ravalba* (Fig. 4), and the next new species, *F.trigonum* sp. nov. (Fig. 2), but it seems that the overall color of the present new species is lighter compared to the three species mentioned above. It can be clearly distinguished from others by the characteristics of male genitalia.

In the male genitalia, *F.qiana* sp. nov. (Figs 7, 8) is most similar to *F.trigonum* sp. nov. (Figs 9, 10), as both have almost flat or beveled cucullus and a large, bristled process at the basal part of the valva. However, *F.qiana* sp. nov. has a pair of stronger and asymmetrical apical spines on the juxta, with the left (when viewed ventrally) spine usually slightly longer than the right; a more elongated and finger-like saccus; a smaller, claw-like, curved cornutus at the base of the vesica; and lacks the triangular sclerite near the base of the vesica. In contrast, *F.trigonum* sp. nov. has a pair of thinner, almost equal-length, finger-like spines on the juxta apically; a shorter, wider, triangular saccus; a thicker and slightly curved cornutus at the base of the vesica; and a triangular sclerite near the base of the vesica. Additionally, *F.qiana* sp. nov. differs from *F.melkaya* (Fig. 11) and *F.ravalba* (Fig. 12) by the latter two having a rounded cucullus on the valva, a small bristled process at the base of the valva, and a shorter, stouter phallus.

#### Description.

**Male. *Adult*.** Forewing length 7.5–8.0 mm, wingspan 16–17 mm (*N* = 4). Antennae filiform, brown. Labial palpi upcurved, pale brown. Head and thorax grayish-white with a tinge of brown; abdomen grayish-white to pare brown. Forewings grayish-white with a series of serrated reddish-brown transverse lines, among which the antemedial line distinct, brown, zigzag; medial and postmedial areas of forewing, and tornus relatively darker, with two large, white, oval spots with black scales around the edges and a series of indistinct small circular spots near tornus, and a small, white, round dot near distal part of cell; postmedian line distinct, forming a broad, indistinct reddish-brown band extending from c. 3/5 of the costal margin from the wing base to tornus. Fringe grayish-white with a tinge of light brown. Hindwings reddish-brown, with dark brown scales mixed in the costal and inner margin areas; fringe light brown.

***Male genitalia*.** Uncus short, pointed apically. Gnathos flat, fish-tail-shaped, comb-like distally. Valva wider at the base; inner side of the cucullus obviously protruding, causing the end of the cucullus to look flat or beveled; basal part of valva with a near-elliptical sclerotized plate, covered with dense, long bristles laterally; sacculus slightly swollen at base. Juxta flat, with a pair of asymmetrical, slender, sclerotized apical spines apically, the left one always slightly longer than the right. Saccus long, broad at the base, gradually narrowing to the middle, then finger-like at distal half part. Phallus slender, tubular, slightly curved, sclerotized at terminal part. Vesica bearing 4–5 cornuti in total, basal one large, hook-shaped; second one small, near the basal cornutus; subapical cornuti 1 or 2 in numbers, apical and subapical cornuti same in size.

***Female genitalia*.** Unknown.

#### Phenology and habitat.

**Figures 16–20.**
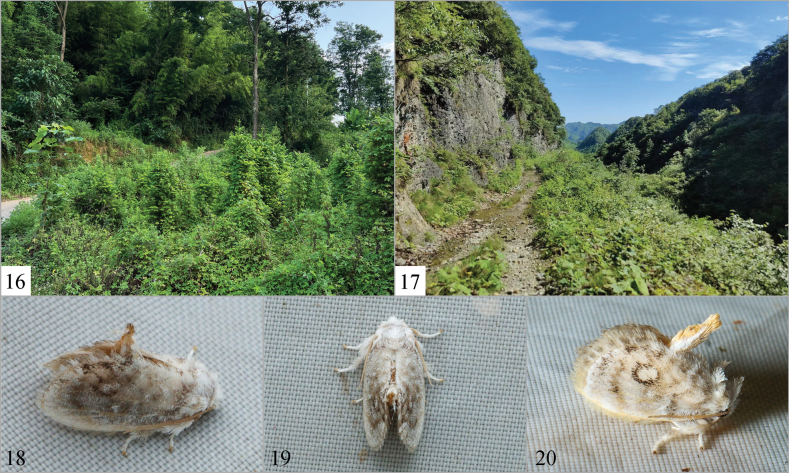
Biotope and living habitus **16, 18–19***F.qiana* sp. nov., the collecting site is Xingren City, Guizhou Province, China; **17, 20***F.trigonum* sp. nov., the collecting site is Zhaotong City, Yunnan Province, China.

The type specimens were collected in June at an altitude of c. 1470 m. The collection site is close to a mixed coniferous and broad-leaved forest, with bamboo forest, bushes, and farmland surrounding it (Fig. 16). The immature stages are still unknown.

#### Distribution.

China (Guizhou).

#### Etymology.

The new species name is derived from the abbreviation of Guizhou Province in China, “Qian”, which is the type locality of *F.qiana* sp. nov.

### ﻿ Fignya trigonum sp. nov.

Taxon classificationAnimaliaLepidopteraLimacodidae

667767C9-D4C4-5081-B738-87C28F44C770

https://zoobank.org/0F6D83BD-C06D-4303-A109-4E3DB4B037D8

Figs 2 , 9 , 10 , 20

#### Type material.

***Holotype***: China • ♂, Yunnan Province, Zhaotong City, Xiaocaoba Town, Yutang Village, 1864 m, 16.vii.2023, RT Xu & MX Han leg., genit. prep. WuJ-1136-1, in NEFU.

***Paratypes***: China • 2♂, same data as for holotype, genit. prep. WuJ-1111-1, 1112-1, all in NEFU.

#### Diagnosis.

*F.trigonum* sp. nov. (Fig. 2) is closest to *F.qiana* sp. nov. (Fig. 1) both in appearance and male genitalia. The differences between the two have been listed in the diagnosis section of *F.qiana* sp. nov. The most significant difference in male genitalia from the other two species, *F.melkaya* (Fig. 11) and *F.ravalba* (Fig. 12), are that the new species has an obviously protruding inner side of the cucullus, a well developed thick and long horn-like cornutus at the base of the vesica, and a triangular sclerite. In contrast, the inner side of the cucullus in *F.melkaya* and *F.ravalba* is smooth, and the developed horn-shaped basal cornutus and triangular sclerite are absent.

#### Description.

**Male. *Adult*.** Forewing length 7.5–8.0 mm, wingspan 16.6–18.0 mm (*N* = 3). Antennae filiform, brown. Labial palpi upcurved, light brown. Head and thorax grayish-white with a tinge of brown; abdomen grayish-white to light brown, terminal with grayish-white to light brown scale tuft. Forewing grayish-brown with a series of serrated reddish-brown to dark brown, serrated transverse lines, among which the antemedial line distinct, dark brown, zigzag; medial and postmedial areas of forewing, and tornus brown to dark brown, with two large, white, oval spots with black scales around the edges and a series of indistinct small circular spots near tornus, and a small, indistinct, white dot located at the end of discal cell; postmedian line grayish-white, extending from c. 3/5 of the costal margin from the wing base to tornus; terminal area black, mixed with some brown and white scales. Fringe grayish-brown with a tinge of dark brown. Hindwings reddish-brown, with dark brown scales mixed in the costal and inner margin areas; fringe pale yellow.

***Male genitalia*.** Uncus short, pointed apically. Gnathos flat, fish-tail-shaped, comb-like distally. Valva narrow and long, swollen at the base, parallel-sided in the middle; inner side of the cucullus slightly protruding; basal part of valva with a near-elliptical, sclerotized plate, which is densely covered with long bristles laterally; sacculus slightly swollen. Juxta flat, with a pair of slender, sclerotized lateral processes. Saccus long, broad at the base, gradually narrowing to the end, rounded terminally. Phallus tube-shaped, sclerotized at terminal part. Vesica with 3 diverticula, bearing 3 cornuti; basal diverticulum with a strongly sclerotized, slightly curved, long, large cornutus, and with a sclerotized triangular sclerite next to it; medial and apical diverticula each with one strongly sclerotized, nail-shaped cornutus.

***Female genitalia*.** Unknown.

#### Phenology and habitat.

The type specimens were collected in July at an altitude of c. 1864 m. The collection site is located in a high mountainous area, surrounded by various types of broad-leaf trees. The ground cover consists of herbs and small shrubs (Fig. 17). The immature stages are still unknown.

#### Distribution.

China (Yunnan).

#### Etymology.

The new species name is derived from the Latin “trigonum”, meaning triangle, referring to a unique sclerotized triangular sclerite at the base of vesica.

### ﻿ Fignya brachygnatha

Taxon classificationAnimaliaLepidopteraLimacodidae

(Wu & Fang, 2008) comb. nov.

00AE9E6B-4161-5A36-936E-888EBDA04973

Figs 6 , 15

Kitanola brachygnatha Wu & Fang, 2008, *Acta Entomol. Sinica* 51 (8): 866. Type locality: China, Yunnan, Xishuangbanna. Holotype: male, in IZCAS.

#### Material examined.

China • ♂, Yunnan Province, Xishuangbanna Dai Autonomous Prefecture, Jinghong City, Mengyang Town, Yexianggu, 4–5.viii.2018, HL Han & MR Li leg., genit. prep. WuJ-243-1, in NEFU.

#### Diagnosis.

The external characters of *F.brachygnatha* (Wu & Fang, 2008) comb. nov. (Fig. 6) are characterized by: the forewing with a grayish-brown ground color, scattered with ochre scales; the antemedial line is ochre-yellow, zigzag; the postmedial line is reddish-brown; at the end of the discal cell, there is a round white spot, below which is a larger white spot surrounded by dark brown scales; and the terminal area is triangular, grayish-white. Hindwings grayish-white, with a slightly darker coloration at the apex.

In the male genitalia (Fig. 15), the uncus is triangular with a small apical spine; the gnathos is short and broad, with a rounded tip; the valva is narrow and elongated, with a broad and rounded cucullus; the juxta is shield-shaped, with a pair of weakly sclerotized finger-like lateral process apically. The phallus is slender and bifurcate at the apex.

#### Distribution.

China (Yunnan).

#### Remarks.

Wu and Fang (2008) placed this species in the genus *Kitanola* Matsumura, 1925, based on its similarity to *K.speciosa* Inoue, 1956 [currently *Mediocampaspeciosa* (Inoue, 1956), see Solovyev 2008: 23]. However, both the forewing pattern and the male genitalia of *F.brachygnatha* comb. nov. show significant differences from those of *Kitanola* species and are more closely aligned with the genus *Fignya*.

*F.brachygnatha* comb. nov. can be distinguished from the type species of the genus *Kitanola*, *K.sachalinensis* Matsumura, 1925 [currently a synonym of *K.uncula* (Staudinger, 1887)], by several morphological features. The newly combined species displays a grayish-brown forewing coloration, characterized by two distinct white circular spots near the end of the discal cell, which are surrounded by dark brown scales. The male genitalia are marked by a spoon-shaped gnathos, an absence of any processes in the valva, and a well-developed saccus. The aedeagus is stout and long, apical half forked, with a pointed apex.

In contrast, *K.uncula* exhibits highly variable forewing coloration, typically presenting 1–2 irregular discal spots. The male genitalia of *K.uncula* are distinguished by a large, hook-shaped gnathos, a valva with a fairly large fold on its inner surface, an inconspicuous saccus, and a curved aedeagus with a large, hook-shaped apical process.

Despite some discrepancies in the male genitalia characteristics compared to typical *Fignya* species, such as a rounded gnathos apex instead of comb-like, and the absence of a basal hairy process on the valva, *F.brachygnatha* shares several key characters with *Fignya*: (1) similar forewing patterns; (2) short uncus with a small apical spine; (3) juxta with a pair of finger-like lateral process at the apex; and (4) the saccus is long. Therefore, we formally transfer this species to the genus *Fignya*.

The newly combined species, as well as *F.samkosa* sp. nov., exhibit some differences in appearance and male genitalia from the rest of the genus as follows (the corresponding characters of the rest of the genus are in brackets): (1) the antemedial line and fringe of the forewing are golden (mostly grayish-brown to reddish-brown); (2) the gnathos is spoon-shaped, with the apical part nearly membranous and rough in surface (the gnathos is fishtail-shaped, with a comb-like tip); (3) the bristled process at the base of the valva is reduced to a small triangular flap or absent (the basal process of valva is well developed and bears laterally large bristles); and (4) the phallus is slender and bifurcated apically (the phallus is relatively short and thick, containing cornuti of varying sizes and numbers in the vesica).

We hypothesize that *F.brachygnatha* comb. nov. and *F.samkosa* sp. nov. may represent a lineage within the genus *Fignya* and might form a sister-group relationship with other congeners in this genus. However, this hypothesis requires further confirmation through molecular analysis.

### ﻿ Fignya samkosa sp. nov.

Taxon classificationAnimaliaLepidopteraLimacodidae

4F03761C-0BB6-575B-AFAF-76521FE9B8D8

https://zoobank.org/90E4D02D-3315-4D83-BA1A-F62A75D65064

Figs 5 , 13 , 14

#### Type material.

***Holotype***: Cambodia • ♂, Samkos, 7–8.ii.2015, YS Bae leg., genit. prep. WuJ-1132-1, in NEFU.

***Paratypes*** • 2♂, same data as for holotype, genit. prep. WuJ-1131-1, all in NEFU.

#### Diagnosis.

The new species (Fig. 5) is most similar to the newly combined species, *F.brachygnatha* comb. nov. (Fig. 6), described in this paper. There is no obvious difference in appearance between these two species, but the male genitalia can be clearly distinguished: *F.samkosa* sp. nov. (Figs 13, 14) has a relatively straight valva, a small triangular hairy flap at the base of the valva, and a slender phallus; whereas the valva of *F.brachygnatha* comb. nov. (Fig. 15) is slightly curved inward, lacks the hairy flap at the base of the valva, and has a thicker phallus. The differences between *F.samkosa* sp. nov. and the other four Fignya species are provided in the remarks section of F.brachygnatha comb. nov.

#### Description.

**Male. *Adult*.** Forewing length 6.0–6.5 mm, wingspan 14–15 mm (*N* = 3). Antennae filiform, brown. Labial palpi upcurved, brown. Head and thorax grayish-white with a tinge of pale brown; abdomen grayish-white to pale brown. Forewings grayish-brown with a series of serrated, golden and reddish-brown transverse lines, among which the antemedial line distinct, golden, zigzag; medial and postmedial lines fuzzy, reddish-brown; discal cell and tornus bear a total of three obvious white spots with black edges. Apex area white, brown on outer edge. Fringe golden. Hindwing reddish-brown; fringe long, pale yellow.

***Male genitalia*.** Uncus narrow and long, pointed apically. Gnathos spoon-shaped, slightly sclerotized and nearly membranous at the end, with densely packed small granular scobination on the surface. Tegument wide. Valva straight, lateral margins nearly parallel; valva with a hairy, small, triangular flap near the base; cucullus rounded. Juxta flat, shield-shaped, upper margin slightly concave in the middle. Saccus long, wide at base, then finger-like. Phallus slender, slightly curved, bifurcated from near the middle, each with a claw-like spine apically.

***Female genitalia*.** Unknown.

#### Phenology and habitat.

The type specimens were collected in February in the Phnom Samkos Wildlife Sanctuary of western Cambodia. The region has a typical tropical monsoon climate, characterized by distinct wet and dry seasons. The sanctuary’s vegetation includes a mix of evergreen forests, montane forests, and bamboo groves. The immature stages are still unknown.

#### Distribution.

Cambodia (Samkos).

#### Etymology.

The new species is named after its type locality, the Phnom Samkos Wildlife Sanctuary in western Cambodia.

### ﻿Key to the species of *Fignya* based on appearance and male genitalia, with distributions

**Table:** 

1	Forewing length 8–9 mm, antemedial line and fringe reddish-brown or dark brown; gnathos fish-tail shaped, comb-like apically; basal part of the valva with a large process which bears laterally large bristles; phallus not bifurcated apically, vesica bears developed cornute	**2**
–	Forewing length 6–7 mm, antemedial line and fringe golden; gnathos spoon-shaped, apically slightly sclerotized and nearly membranous, with densely packed small granular scobination on the surface; basal process of the valva small or absent; phallus bifurcated apically, vesica without cornutus	**5**
2	Juxta with a pair of developed, spin-like lateral processes apically; phallus without long spines on the surface	**3**
–	Juxta without lateral processes apically; phallus bears 3–5 strongly sclerotized, long spines on the surface	***F.ravalba*** (China: Xizang)
3	Basal process of the valve large, the inner side of the cucullus is obviously protruding (causing the end of the cucullus to look flat or beveled), and the cornuti at the base of the vesica is well developed	**4**
–	Basal process of the valve slightly smaller, the inner side of the cucullus is smooth (making the cucullus looks more blunter and more rounded), and the cornuti in the vesica are almost equal in size	***F.melkaya*** (Vietnam: Mt. Fan-si-pan (West); China: Sichuan)
4	Cornutus at the base of the vesica short, hook-shaped, without triangular sclerite	***F.qiana* sp. nov.** (China: Guizhou)
–	Cornutus at the base of the vesica long, slightly curved, with a triangular sclerite next to it	***F.trigonum* sp. nov.** (China: Yunnan)
5	Valve straight, with a small, triangular, hairy flap near the base; the phallus is thin	***F.samkosa* sp. nov.** (Cambodia: Samkos)
–	Valve slightly inward-curved, without processes on the surface; the phallus is relatively thick	***F.brachygnatha* comb. nov.** (China: Yunnan)

## Supplementary Material

XML Treatment for Fignya

XML Treatment for Fignya qiana

XML Treatment for Fignya trigonum

XML Treatment for Fignya brachygnatha

XML Treatment for Fignya samkosa

## ﻿Additional information

### Author ORCIDs

Jun Wu https://orcid.org/0000-0002-8032-2522

Huilin Han https://orcid.org/0000-0002-2045-6182

### Data availability

All of the data that support the findings of this study are available in the main text
